# An Investigation into the Strength of the Association and Agreement Levels between Subjective and Objective Sleep Duration in Adolescents

**DOI:** 10.1371/journal.pone.0072406

**Published:** 2013-08-09

**Authors:** Teresa Arora, Emma Broglia, Dunstan Pushpakumar, Taha Lodhi, Shahrad Taheri

**Affiliations:** 1 School of Clinical and Experimental Medicine, University of Birmingham, Birmingham, United Kingdom; 2 Birmingham and Black Country National Institute for Health Research Collaboration for Leadership in Applied Health Research and Care, University of Birmingham, Birmingham, United Kingdom; 3 School of Health and Population Sciences, University of Birmingham, Birmingham, United Kingdom; 4 School of Medicine, University of Manchester, Manchester, United Kingdom; University of Rochester, United States of America

## Abstract

**Study Objectives:**

The majority of adolescent sleep research has utilized self-reported sleep duration and some have based information on a solitary question. Whilst some have claimed to have validated sleep survey data with objective actigraphy measures in adolescents, the statistical approach applied only demonstrates the strength of the association between subjective and objective sleep duration data and does not reflect if these different methods actually agree.

**Methods:**

Data were collected as part of the Midlands Adolescents Schools Sleep Education Study (MASSES). Adolescents (n=225) aged 11-13 years provided estimates for weekday, weekend and combined sleep duration based on self-reported survey data, a 7-day sleep diary, and wrist-worn actigraphy.

**Results:**

We assessed the strength of the relationship as well as agreement levels between subjective and objectively determined sleep duration (weekday, weekend and combined). Subjective diary sleep duration was significantly correlated with actigraphy estimates for weekday and weekend sleep duration r=0.30, p≤0.001 and r=0.31, p≤0.001 respectively. Pitman’s test demonstrated no significant difference in the variance between weekend sleep duration (r=0.09, p=0.16) and combined sleep duration (r=0.12, p=0.08) indicating acceptable agreement between actigraphy and sleep diary sleep duration only. Self-reported sleep duration estimates (weekday, weekend and combined) did not agree with actigraphy determined sleep duration.

**Conclusions:**

Sleep diaries are a cost-effective alternative to survey/questionnaire data. Self-reported measures of sleep duration in adolescents do not agree with actigraphy measures and should be avoided where possible. Previous adolescent sleep studies that have utilized self-reported survey data may not provide a complete representation of sleep on the outcome measure of interest.

## Introduction

Daytime sleepiness and circadian phase delay in adolescents is widespread and is likely to be the result of insufficient sleep duration, particularly during the week. Involuntary sleep restriction in adolescents is likely to occur from 1) physiological alterations associated with puberty, known to delay sleep onset [1]; and 2) early school attendance, forcing adolescents to rise prematurely [2] despite preceding delays in sleep onset. Sleep debt, commonly accrued during the week, is usually repaid during weekends/holidays, where longer sleep duration and later wake times are permitted [3]. Inadequate sleep duration in adolescents has been previously associated with increased body mass index [4,5], poorer academic performance [6], substance use [7], and psychological status [7].

Whilst evidence for the adverse effects of sleep loss in adolescent populations is prolific, most studies have relied upon self-reported sleep duration. This may be problematic and misrepresent the overall understanding of adolescent sleep in relation to various outcome measures. For example, one study assessed three sleep measures in adolescents (parental report, actigraphy and 8-day self-reported sleep diary). Compared to actigraphy estimates, parental reports and sleep diary estimates were +120 minutes and +85 minutes, respectively [8], leading the authors to suggest that actigraphy estimated sleep duration might be under-estimated. Other studies have utilized self-reported adolescent sleep duration based on a solitary sleep question [9,10].

Self-reported sleep duration may produce inaccurate estimates due to multiple biases including recall, social desirability and/or reporting of time in bed rather than sleep duration per se. Sleep diaries, providing they are completed daily, may be a more effective method compared to asking adolescents to report average sleep duration relating to the previous month. Whilst some have employed objective sleep duration measures (polysomnography [11] and actigraphy [12]), these methods are less well utilized, possibly due to financial implications.

Previous work has shown good agreement for actigraphic measured sleep onset and offset with sleep diaries in children [13]. Similar results have been reported for adolescents where self-reported sleep duration (Schools Sleep Habits Survey and sleep diary) estimates were positively and significantly associated with actigraphy measures [14]. Others have demonstrated only a weak correlation between sleep diaries and self-reported sleep duration, which produced different effects on the outcome measure [10]. Correlation techniques, however, only demonstrate the strength of the relationship between two measures but do not reflect if the two techniques agree. Bland and Altman recommend plotting the difference between objective and subjective measurements against the mean to assess if two measures agree [15]. We therefore investigated the strength of the relationship as well as the level of agreement between three sleep duration measures (actigraphy, sleep diary and self-report) in a large sample of United Kingdom (UK) adolescents. Given the difference in sleep duration between weekdays and weekends in adolescents, we assessed average 1) weekday sleep duration; 2) weekend sleep duration and 3) combined sleep duration for all three measures.

## Methods

### Ethics statement

The University of Birmingham, Research and Commercial Services (ERN_08-437) granted ethical approval for the study. Written informed consent was sourced from all parents and student participants. The investigation was conducted according to the principles expressed in the Declaration of Helsinki. A total of 301 students, registered at one of eight secondary schools, were invited to participate in the Midlands Adolescent Schools Sleep Education Study (MASSES), previously described [5]. A variety of school types were included: secondary (64.5%), grammar (10.2%) and independent (25.3%). All participating students were in Year 7 (70.9%) or Year 8 (29.1%) of secondary education, aged 11-13 years (12±0.7), comprising 42.7% boys. Participants were excluded if 1) they had travelled to a different time zone 4 weeks prior to providing data; 2) had a diagnosed sleep disorder(s); 3) were taking prescribed sleep medication; 4) did not have written parental consent; and/or 5) did not provide personal written consent. All data were collected March–June 2012.

### Sleep duration

Self-reported sleep duration (minutes) for weekdays and weekends was obtained from the Schools Sleep Habits Survey (SSHS) by asking: “Work out how long you USUALLY sleep on a normal school/weekend night. Do not include the time you spend awake in bed”.

Following completion of the SSHS, participants were issued with wrist actigraphy (GT3X+, Actigraph, Florida, USA), previously validated against sleep-wake polysomnography [16]. Participants were instructed to wear the device on their non-dominant wrist for 7 consecutive days alongside completing a daily sleep diary. Average weekday and weekend sleep duration (minutes) was calculated from the sleep diary by noting the difference between the time the participant thought they fell to sleep and the time they woke up the following morning. Actigraphy data were downloaded and estimated sleep duration and wake after sleep onset (WASO) was obtained according to the manufacturers predefined algorithms. Sleep diary estimates were then checked against the downloaded actigraphy data for each participant, as recommended by Acebo et al [17]. Average diary and actigraphy weekday sleep duration was calculated by adding sleep duration for Sunday through to Thursday and then divided by five. Average diary and actigraphy weekend sleep duration was derived by adding sleep duration for Friday and Saturday and then divided by two. Average combined sleep duration was calculated by adding all sleep duration data points together and then dividing by the appropriate number (sleep diary and actigraphy). Average combined self-reported sleep duration, obtained from the SSHS, was calculated by 1) multiplying weekday sleep duration by five; 2) multiplying weekend sleep duration by two; and 3) dividing the total amount by seven.

### Statistical analyses

All statistical analyses were performed using Stata version 12 (Texas, USA). Data were assessed for normality of distribution for each of the three sleep duration estimates. Scatterplots and Pearson’s correlations were performed for each of the three measures to assess the potential extent of the associations between sleep duration for 1) actigraphy and self-report; 2) actigraphy and sleep diary; 3) sleep diary and self-report for each of the following: a) average combined sleep duration; b) average weekday sleep duration; and c) average weekend sleep duration. P values were adjusted for multiple comparison tests through the Bonferroni correction. The correlation coefficient was also obtained to assess the relationship between WASO and average number of night awakenings from the sleep diary. One-way ANOVA and Bonferroni post hoc tests were performed to assess mean differences between the three measures for average weekday, weekend and combined sleep duration. Bland-Altman plots and statistical information relating to levels of agreement for the above were then conducted. Elements of the Bland-Altman plot include: y-axis, which represents the difference between the two measurements being assessed; x-axis, which represents the average of the two methods; a horizontal line, which represents the bias; two horizontal lines representing the 95% Limits of Agreement; Limits of Agreement define the range in which 95% of the differences between methods are expected to fall and are calculated as the bias±1.96 standard deviation. A classical test of variance for paired samples based on the bivariate normal distribution that compares the variance of the difference with the variance of the average is calculated according to the Pitman’s test, which we also report concurrent with each Bland-Altman plot. We also computed, through multilevel modeling, intra-class correlations to assess variance of the data, according to school effects, for each of the three sleep measures for combined average sleep duration.

## Results

**Figure 1:**
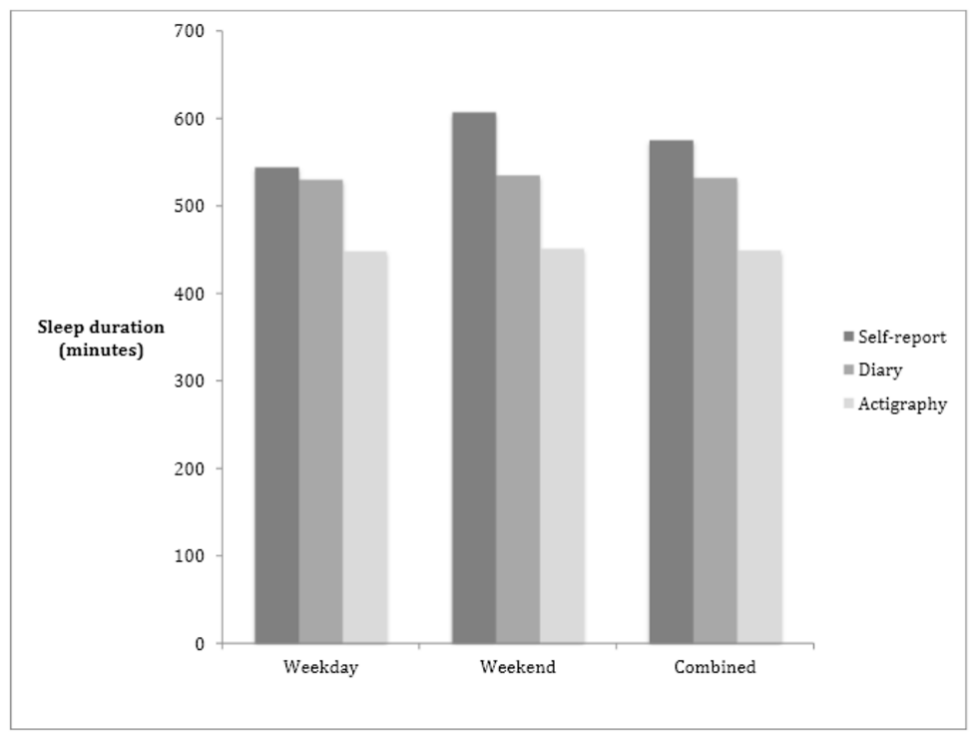
Mean sleep duration for each sleep measure and each time point in 225 adolescents.

Of the 301 participants, information on all variables of interest was available for 75% (n=225). There were no significant gender differences between those whose data were included in our analyses and those who were not (p>0.05). Figure 1 shows the mean sleep duration for each of the three measures, for weekday, weekend and combined. Mean±standard deviation for weekday sleep duration (minutes) estimated by self-report, sleep diary and actigraphy were 544±64, 530±48, and 448±42, respectively (F=220.34, p<0.001). Weekend sleep duration estimated by self-report, sleep diary and actigraphy were 607±98, 535±59, and 451±54 minutes, respectively (F=259.27, p<0.001). Combined sleep duration (minutes) estimated by self-report, sleep diary and actigraphy were 575±66, 531±44, and 449±39, respectively (F=358.30, p<0.001). Details of the Bonferroni post hoc tests are shown in Table 1. The ICC, computed from multilevel models, showed that 21% of the variation for combined average sleep duration (sleep diary) was attributed to school effects, almost 13% for actigraphy, but only 7% of the variation was attributed to schools for self-reported data.

**Table 1:** Mean differences between the three sleep measures for average weekday, weekend and combined sleep duration in 225 adolescents.

**Measure**	**Measure**	**Mean difference**	**p value**	**95% CI**
**Average weekday sleep duration (minutes)**				
Self-report	Sleep diary	13.6	0.017	1.8, 25.4
Self-report	Actigraphy	95.5	<0.001	83.7, 107.3
Actigraphy	Sleep diary	-81.9	<0.001	-93.7, -70.1
**Average weekend sleep duration (minutes)**				
Self-report	Sleep diary	72.0	<0.001	55.4, 88.5
Self-report	Actigraphy	156.7	<0.001	140.2, 173.3
Actigraphy	Sleep diary	-84.8	<0.001	-101.3, -68.2
**Average combined sleep duration (minutes)**				
Self-report	Sleep diary	57.6	<0.001	44.8, 70.4
Self-report	Actigraphy	111.8	<0.001	99.0, 124.6
Actigraphy	Sleep diary	-54.2	<0.001	-67.0, -41.3

CI = confidence intervals

Correlation coefficients for each of the relationships assessed are shown in Table 2. In brief, weekend sleep duration was significantly correlated in all combinations. Self-reported sleep duration was not significantly correlated with actigraphy sleep duration for weekday or combined sleep duration. Sleep diary estimated sleep duration was significantly correlated with actigraphy for weekday, weekend and combined sleep duration. A positive association was also observed between WASO and average number of night awakenings (diary) r=0.14, p=0.04.

**Table 2:** Correlation coefficients for average weekday, weekend and combined sleep duration according to self-report, sleep diary and actigraphy in 225 adolescents.

Average weekday sleep duration	Sleep diary	Actigraphy
Self-reported sleep duration	0.48**	0.17
Sleep diary sleep duration		0.30**
Average weekend sleep duration		
Self-reported sleep duration	0.21*	0.02
Sleep diary sleep duration		0.31**
Average combined sleep duration		
Self-reported sleep duration	0.41**	0.06
Sleep diary sleep duration		0.31**

Data are presented as correlation coefficients.

* p≤0.01; **p≤0.001 (adjusted for multiple test comparisons through Bonferroni correction).

**Figure 2:**
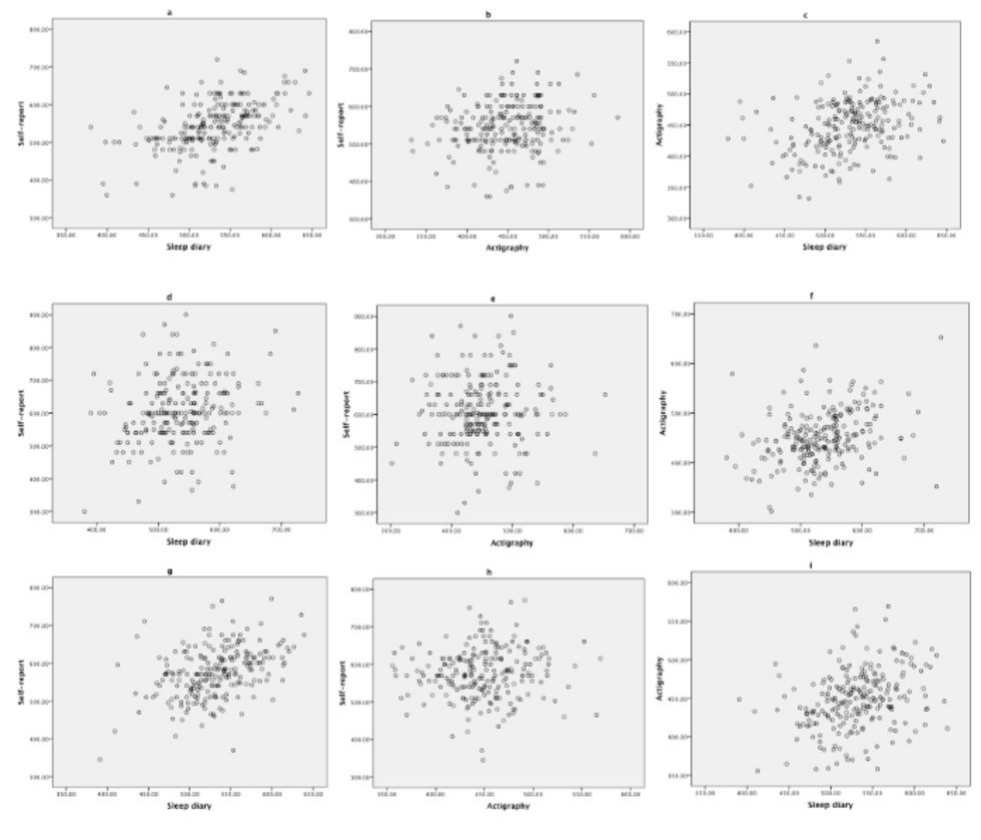
A series of scatterplots demonstrating the relationships for the average weekday, weekend and combined sleep duration for each of the three sleep measures in 225 adolescents. a: relationship between weekday self-report and sleep diary sleep duration. b: relationship between weekday self-report and actigraphy sleep duration. c: relationship between weekday actigraphy and sleep diary sleep duration. d: relationship between weekend self-report and sleep diary sleep duration. e: relationship between weekend self-report and actigraphy sleep duration. f: relationship between weekend actigraphy and sleep diary sleep duration. g: relationship between combined self-report and sleep diary sleep duration. h: relationship between combined self-report and actigraphy sleep duration. i: relationship between combined actigraphy and sleep diary sleep duration.

**Figure 3:**
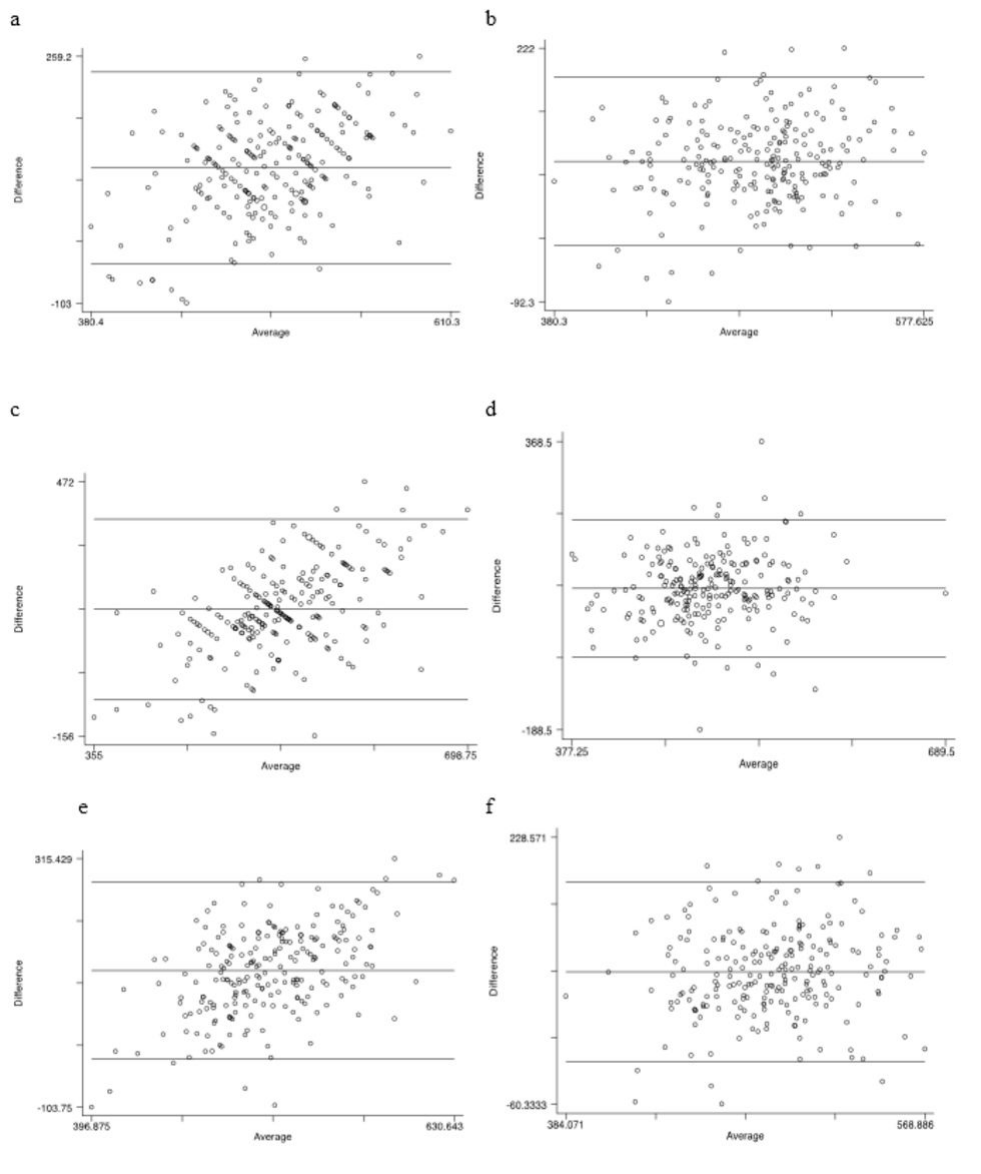
A series of Bland-Altman plots to assess the limits of agreement between self-report and sleep diary with actigraphy according to weekday, weekend and combined sleep duration in 225 adolescents. a: Weekday self-report versus actigraphy sleep duration. b: Weekday sleep diary versus actigraphy sleep duration. c: Weekend self-report versus actigraphy sleep duration. d: Weekend sleep diary versus actigraphy sleep duration. e: Combined self-report versus actigraphy sleep duration. f: Combined sleep diary versus actigraphy sleep duration.

Figure 2 shows the data plotted for each of the associations assessed (a-c = weekday; d-f = weekend; g-i = combined). A series of Bland-Altman plots are shown in Figure 3 assessing the levels of agreement between self-reported and sleep diary with actigraphy for weekday, weekend and combined sleep duration. Table 3 details the range of the limits of agreement, mean difference between the sleep duration measures assessed along with 95% confidence intervals (CI), range of sleep duration (minutes), Pitman’s variance between the two measures assessed along with the p value. The mean difference was greater and well above zero for self-reported sleep duration compared to actigraphy for weekday, weekend and combined sleep duration: 95.47, 156.74 and 126.80 minutes, respectively.

**Table 3:** Levels of agreement for self-report and sleep diary against actigraphy for weekday, weekend and combined sleep duration according to Bland-Altman analyses.

Limits of agreement	Mean difference (95% CI)	Range (minutes)	Variance (r)	p value
Weekday self-reported vs. actigraphy sleep duration				
-45.45-236.40	95.47 (86.22-104.73)	380.40-610.30	0.41	<0.001
Weekday sleep diary vs. actigraphy sleep duration				
-22.49–186.22	81.86 (75.01-88.72)	380.30-577.63	0.14	0.03
Weekend self-reported vs. actigraphy sleep duration				
-65.78–379.27	156.74 (142.13-171.36)	355.00-698.75	0.54	<0.001
Weekend sleep diary vs. actigraphy sleep duration				
-47.69–217.21	84.76 (76.06-93.46)	377.25-689.50	0.09	0.16
Combined self-reported vs. actigraphy sleep duration				
-22.84–276.43	126.80 (116.97-136.63)	396.88-630.64	0.49	<0.001
Combined sleep diary vs. actigraphy sleep duration				
-14.15–180.09	82.97 (76.59-89.35)	384.07-568.89	0.12	0.081

Variance was determined by the Pitman’s test of difference in variance between the two measures of sleep duration assessed.

## Discussion

Our study is the first to investigate the strength of the relationships and agreement levels between weekday, weekend and combined average sleep duration for three sleep measures (self-report, sleep diary and actigraphy) in young adolescents. We observed several significant correlations between two subjective sleep duration measures and objectively determined actigraphy sleep. On closer inspection, through deployment of Bland-Altman plots and Pitman’s test for variance, we found sleep diary and actigraphy (average weekend and combined sleep duration) did not have a significant variance, indicating an acceptable level of agreement for these two measures of sleep duration in young adolescents.

Our findings revealed that both self-reported and sleep diary sleep duration was longer than actigraphy sleep duration, consistent with others [8,13,18]. Over-estimation of sleep duration in adolescents is likely to be the result of reporting time in bed and/or disregarding the amount of time spent awake in bed. Sleep diary reports, were nearer to actigraphy sleep duration compared to survey self-reports. Self-reported survey data typically request sleep duration based on the previous two weeks at the time of responding. This method requires the participant to generalize across the specified time period to produce an estimate, which may be subject to multiple biases. A sleep diary may, however, provide a more accurate representation if it is completed daily and within short proximity to waking. Our findings demonstrate significant correlations between weekday, weekend and combined sleep duration for sleep diary and actigraphy as well as for self-reported sleep duration and sleep diary reports. We were, however, only able to confirm a good level of agreement between sleep duration estimates from sleep diary and actigraphy for weekend and combined sleep duration, indicating that the sleep diary method is preferable to survey reported sleep duration. Whilst the sleep diary method is a good cost-effective alternative and can be administered to large samples, response rates may be reduced along with a higher risk of missing/incomplete data.

An array of adolescent sleep studies, examining various outcomes, have utilized self-reported sleep duration based on survey data [3,6,7,19–22]. Indeed, some have deployed a solitary question [9,10]. Based on our findings, if sleep duration in these studies have been over-estimated by approximately one hour then conclusions drawn from these studies may be inaccurate and subject to systematic bias. For example, if respondents reported achieving 9 hours of sleep, which is recommended for adolescents [23], but they actually gain ≤8 hours of sleep then this may mean that the effect size may be minimized compared to what is reported. To illustrate, Sun and colleagues reported a significant and negative association between sleep duration and overweight in a large sample (n=5,753) of 12-13 year olds [24]. Compared to girls who reported sleeping for ≥10 hours, those who reported sleeping for <7 hours or 7-8 hours had an 81% and 37% increased risk of overweight, respectively. Assuming these reports of sleep are over-estimated by just one hour, although our data along with others have shown higher disparities, then this would suggest that 1) 6-7 hours, rather than 7-8 hours of sleep, would be associated with a 37% increased risk of overweight; and 2) <6 hours instead of <7 hours of sleep would be associated with an 81% increased risk of overweight compared to those sleeping for ≥9 hours. Thus, the effects are minimized compared to those originally reported.

Previously, attempts have been made to assess different methods of sleep duration in relation to body mass index (BMI) in adolescents (10-19 years old). Knutson and Lauderdale utilized 24-hour time diaries as well as self-reported habitual sleep duration [10]. The authors hypothesized that the two sleep duration measures would be well correlated and that the time diary data would be more accurate and therefore have a stronger association with overweight, as determined by BMI z-score. Interestingly, mean sleep duration from the time diary was calculated as 8.8 hours (weekdays) but only 8 hours based on self-report. The two measures were not well correlated (r=0.27, p<0.001) and time diary data was not significantly associated with overweight whilst self-reported sleep duration was, although the relationship was not linear. It is important to note that time diary data were obtained for just one 24-hour period for one randomly selected weekday and one for the weekend. A 7-day diary would have provided additional information and potentially produced a clearer representation of adolescent sleeping habits. Whilst Knutson and Lauderdale surprisingly observed longer sleep duration from diary data compared to self-reported sleep duration, it is impossible to determine if these reports are accurate in the absence of objective measures.

Whilst our study benefits from a large sample of young adolescents, it is important to acknowledge several limitations. Firstly, self-reported sleep duration was based on one question relating specifically to weekday and weekend, although this is approach is not uncommon and has been used by others [9,10]. Secondly, our data were collected in young adolescents (11-13 year olds) and whilst the gender mix was well balanced, our findings may not be representative of all adolescents or the general population. Thirdly, whilst the wrist-worn actigraphy device has been previously validated against polysomnography (PSG) [16], it is not considered the gold-standard technique for assessment of sleep. However, actigraphy is less invasive and more cost-effective than PSG and may allow individuals to sleep more comfortably and naturally in their own environment in the absence of intrusive equipment. Finally, our results demonstrated that only 7% of the variation was attributable to schools showing individual variation consisted of more than 93% of the total variation for self-reported sleep measures. Variation of sleep diaries and actigraphy were, however, above 8%.

In conclusion, future studies that intend to assess sleep duration in adolescents in relation to any outcome measure should aim to avoid self-reported sleep duration information, but instead incorporate the use of 7-day sleep diaries, unless actigraphy or PSG is available. Sleep diaries and actigraphy provide more detailed information that can be utilized to better inform the outcome of such studies. Previous studies that have employed self-reported sleep duration may have produced inaccurate findings and over-estimated effect sizes.
